# A comparison of foam rolling and vibration foam rolling on the quadriceps muscle function and mechanical properties

**DOI:** 10.1007/s00421-021-04619-2

**Published:** 2021-02-26

**Authors:** Marina Maren Reiner, Christoph Glashüttner, Daniel Bernsteiner, Markus Tilp, Gael Guilhem, Antonio Morales-Artacho, Andreas Konrad

**Affiliations:** 1 Institute of Human Movement Science, Sport and Health, University of Graz, Mozartgasse 14, 8010 Graz, Austria; 2 French Institute of Sport (INSEP), Laboratory Sport, Expertise and Performance (EA 7370), Paris, France

**Keywords:** Muscle stiffness, Shear wave elastography, Foam rolling, Vibration foam rolling, Self-myofascial release

## Abstract

**Purpose:**

The purpose of the study was to investigate the effects of using a vibration foam roll (VFR) or a non-vibration foam roll (NVFR) on maximum voluntary isometric contraction peak torque (MVIC), range of motion (ROM), passive resistive torque (PRT), and shear modulus.

**Methods:**

Twenty-one male volunteers visited the laboratory on two separate days and were randomly assigned to either a VFR group or a NVFR group. Both interventions were performed for 3 × 1 min each. Before and after each intervention, passive resistive torque and maximum voluntary isometric contraction peak torque of the leg extensors were assessed with a dynamometer. Hip extension ROM was assessed using a modified Thomas test with 3D-motion caption. Muscle shear modulus of the vastus lateralis (VL), vastus medialis (VM), and rectus femoris (RF) was assessed with shear wave elastography (SWE).

**Results:**

In both groups (VFR, NVFR) we observed an increase in MVIC peak torque (+ 14.2 Nm, + 8.6 Nm) and a decrease in shear modulus of the RF (− 7.2 kPa, − 4.7 kPa). However, an increase in hip extension ROM (3.3°) was only observed in the VFR group. There was no change in PRT and shear modulus of the VL and VM, in both the VFR group and the NVFR group. Our findings demonstrate a muscle-specific acute decrease in passive RF stiffness after VFR and NVFR, with an effect on joint flexibility found only after VFR.

**Conclusion:**

The findings of this study suggest that VFR might be a more efficient approach to maximize performance in sports with flexibility demands.

## Introduction

Foam rolling is a popular warm-up and/or recovery technique in sports and physical therapy (Pearcey et al. 2015; de Benito et al. 2019). There is evidence that a single bout of foam rolling acutely increases range of motion (ROM) (Bradbury-Squires et al. 2015; Phillips et al. 2018). Five seconds of foam rolling of the hamstring muscles has been found to lead to an acute increase in hip flexion ROM (Sullivan et al. 2013). By comparing even longer durations of foam rolling, Bradbury-Squires et al. (2015) reported similar increases in ROM following 5 × 20 s and 5 × 60 s foam rolling on the quadriceps femoris muscle, indicating a possible saturation effect in ROM changes following a certain bout of foam rolling.

The studies that have focused on the impact of a single foam rolling bout on muscle force have reported contradictory findings. While some studies have reported an increase in muscle strength (Romero-Moraleda et al. 2019) others have either reported no change (Sullivan et al. 2013; Baumgart et al. 2019) or a decrease in muscle strength (Phillips et al. 2018). As these contradictory results cannot be explained by different foam rolling durations, it is reasonable to assume that the effects may originate from other parameters that influence the application, such as the rolling pressure and rolling frequency.

In recent years, several studies have tried to investigate if a single set of vibration foam rolling (VFR) might have a more pronounced effect on various muscle performance parameters than a non-vibration foam rolling (NVFR) exercise. Vibration therapy can stimulate more muscle receptors in three afferent fiber types (Ia, II and Ib), which leads to an increase in motor fiber recruitment (Fallon and Macefield 2007; Germann et al. 2018). It has also been suggested that VFR might have a more pronounced effect on muscle flexibility and muscle mechanical property parameters than a bout of NVFR. Some studies (Sağiroğlui 2017; Lee et al. 2018; de Benito et al. 2019) have reported an increase in hip flexion ROM after both VFR and NVFR, with no difference in the amount of ROM increase between VFR and NVFR. Nevertheless, other studies (Lee et al. 2018; Romero-Moraleda et al. 2019) have reported an increase in ROM with VFR but not with NVFR. Furthermore, with regard to muscle performance, a significant difference between VFR and NVFR effects on hamstring strength has been reported (Lee et al. 2018). However, no difference in countermovement jump height (Sağiroğlui 2017) or quadriceps muscle strength (Lee et al. 2018) has been found. A recent review (Wilke et al. 2020) reported that VFR might be more efficient than NVFR; however, to date, there is not enough evidence to generalize this assumption. It has also been suggested that the possibly superior effect of VFR compared to NVFR might be due to the greater contribution of the mechanoreceptors at higher vibration frequencies (Behm and Wilke 2019). Pacinian corpuscles are sensitive to high-frequency vibrations, and in combination with Ruffini receptors (which are sensitive to lateral stretch and tangential forces), there might be an induced muscle relaxation due to an inhibition of sympathetic activation (Wu et al. 1999; Behm and Wilke 2019).

In addition to functional parameters such as ROM or muscle strength, it has also been investigated if muscle mechanical properties (e.g. muscle stiffness) might explain the changes in muscle–tendon function following a single bout of NVFR. Since several studies of the acute effects of stretching have reported mechanical (reduced muscle stiffness; Konrad et al. 2017) or neuromuscular (reduced pain sensitivity i.e. stretch tolerance; Magnusson et al. 1996) changes as a mechanism for a change in ROM or muscle strength, it can be assumed that similar mechanisms may be responsible for the acute effects following a single bout of foam rolling. The magnitude of the effects of foam rolling are comparable to the effects caused by stretching interventions (Wilke et al. 2020). If a reduced muscle stiffness is supposed to induce an increase in ROM, this change will decrease the passive resistive torque (PRT) (Konrad et al. 2019). Monte and Zignoli (2021) could recently show that muscle stiffness is positively related to rate of force development. Hence, a possible change of muscle stiffness following a bout of foam rolling with or without vibration might affect force production. While some studies have reported a decrease in muscle stiffness following a single set of (non-vibration) foam rolling [Morales-Artacho et al. 2017; Baumgart et al. 2019 (for rectus femoris)], others have reported no changes in muscle stiffness [Baumgart et al. 2019 (for gastrocnemius medialis); Mayer et al. 2019]. Overall, there is little known about the effects of NVFR on muscle mechanical properties. Moreover, to the best of our knowledge, it is not known if a single bout of VFR affects muscle mechanical properties.

Furthermore, to date, no study has compared the effects of VFR and NVFR on both functional parameters [i.e. ROM, maximum voluntary isometric contraction (MVIC)] and the muscle mechanical properties (i.e. muscle stiffness) of the quadriceps femoris muscle. Therefore, the aim of the present study was to compare the effects of a single 3-min bout of NVFR and a 3-min bout of VFR of the quadriceps muscles on hip extension ROM, passive resistive torque (PRT), MVIC, and muscle stiffness [shear modulus of the vastus medialis (VM), vastus lateralis (VL), and rectus femoris (RF)]. We hypothesized an increase in ROM and MVIC and a decrease in PRT and muscle stiffness following both treatments (VFR vs NVFR). However, we expected greater changes with VFR.

## Materials and methods

### Experimental design

**Fig. 1:**
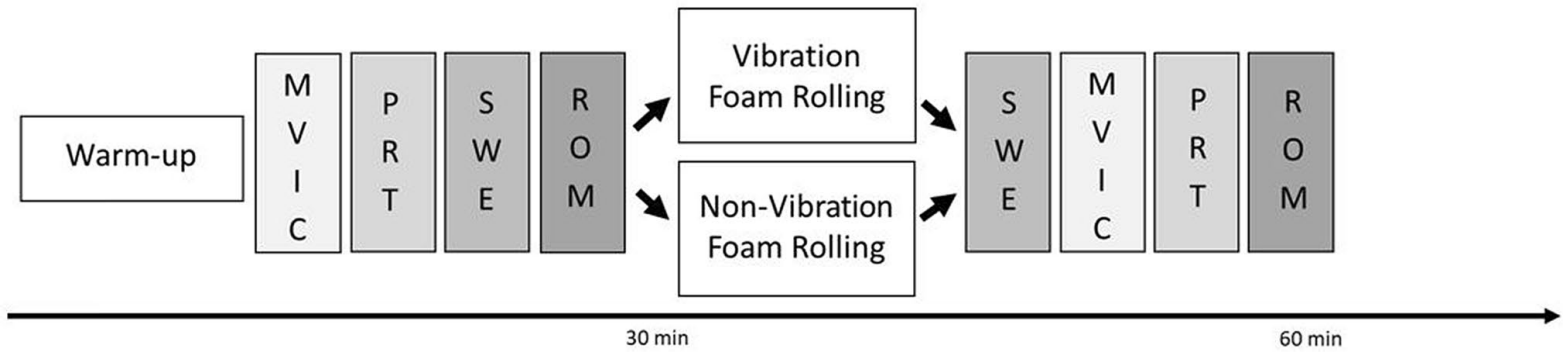
Overview of the experimental design. Maximum voluntary isometric contraction (MVIC)—to measure muscle force; passive resistive torque (PRT)—to measure passive resistance in the muscle; shear wave elastography (SWE)—to measure muscle shear modulus; range of motion (ROM)—to measure maximum joint position

Participants were asked to visit the laboratory on two separate sessions to complete both interventions (VFR vs NVFR) within 2–7 days. Prior to the first measurements, participants had a familiarization session to introduce them to the foam rolling procedure. The intervention was randomized by the participants choosing a hidden card. At both appointments, participants performed a 10-min warm-up on a stationary bike (Monark, Ergomedic 874 E, Sweden) at 60 rev.min^−1^ and a resistance of 90 W. Functional parameters (i.e. ROM, MVIC, PRT) and muscle mechanical properties (i.e. shear modulus) of the right quadriceps muscles were examined pre and post the VFR and NVFR interventions. The duration between the warm-up and the first measurement was about 5 min. The functional parameters were MVIC, PRT, and hip extension ROM. The mechanical properties of the quadriceps muscle were tested in the VL, VM, and RF by shear wave elastography (SWE). The surface electromyography (Myon 320, Myon AG, Zurich, Switzerland) was measured on the VL muscle during MVIC, PRT, and SWE testing, before and after the intervention. Tests were performed in the order and time frame listed in Fig. 1. The order for the SWE was VL, VM, and RF.

### Participants

An a priori sample size calculation (primary outcome variable range of motion) for a repeated-measures ANOVA based on the literature (Phillips et al. 2018) suggests a necessary group size of at least 15 participants (alpha = 0.05, beta = 0.8, *f* = 0.4). Thus, 21 physically active male participants (age: 25.2 ± 3.8 years; weight: 77.6 ± 8.8 kg; height: 182.5 ± 6.9 cm) volunteered in this study. Participants were free of any injuries of the lower extremities. Participants were informed about the test procedure before they signed a written informed consent form. The ethical approval was obtained by the ethical commission of the university and conformed to the standards of the Declaration of Helsinki.

### Procedures

#### Maximum voluntary isometric contraction (MVIC) peak torque

The MVIC measurements were performed with an isokinetic dynamometer (Con Trex MJ, CMV AG, Dübendorf, Switzerland). The participant was seated on the dynamometer, with the hip and knee angle of the right leg (test leg) being 110° (180° = full hip and knee extension) (Jakobsen et al. 2012; Kaya et al. 2019). A custom-made laser device was used to align the center of rotation of the dynamometer with the knee joint axis in a relaxed state right at 110° knee angle. To ensure the same sitting position during all assessments on the dynamometer, we recorded the exact position of the participant during the first MVICs, and we placed the participant in the same position in the following measurements. The trunk and test leg were fixed with straps to minimize the possibility of evasive movement. The lever arm fixation was set about 2 cm above the medial malleolus (Morales-Artacho et al. 2017). Each participant was asked to cross their arms in front of their chest and to perform three MVICs for 5 s each. The participant was asked to push as hard as possible, and received strong verbal encouragement during the measurements. Maximum torque value was communicated after each attempt and participants were motivated to try to exceed the previous result. Between the three MVICs, the participant rested for 1 min. The attempt with the highest torque value was considered for further analysis.

#### Passive resistive torque (PRT)

PRT measurement was done in the same sitting position as previously described for the MVIC measurement. The knee joint was passively moved for five cycles at an angular velocity of 5°/s from 90° to 60°. According to previous studies (Kubo et al. 2002; Mahieu et al. 2009), the velocity of the dynamometer was set to 5°/s to exclude any reflexive muscle activity. Participants were asked to relax completely. The lowest torque value of the last three circles in the extension phase was taken for further analysis.

#### Muscle shear modulus

Muscle shear modulus was measured on the VL, RF, and VM by SWE with an ultrasound scanner (Aixplorer V6, Supersonic Imaging, Aix-en-Provence, France) coupled with a linear transducer array (4–15 MHz, SuperLinear 10–2; Vermon, Tours, France). The machine was used in shear wave elastography mode (musculo-skeletal preset, penetration mode, smoothing level 5, persistence off, scale 0–300 kPa). The measuring system generates a two-dimensional map of the shear modulus of the measured tissue at 1 Hz, with a spatial resolution of 1 × 1 mm. Muscles were scanned using a handheld technique, based on previous studies that allowed a reliable measure for muscle stiffness (Bercoff et al. 2004; Lacourpaille et al. 2012; Hug et al. 2015). The participant was seated on the dynamometer with a hip angle of 110° and knee angle of 70° to achieve a slightly stretched position of the quadriceps muscles (Lacourpaille et al. 2017). To ensure similar placement of the probe in all measurements, reusable foil marked with the scars and birthmarks of the participant’s skin was used, which was also marked with the probe placement. Moreover, to facilitate reproduction during the following measurements, a B-Mode ultrasound image of the measured muscle part was recorded. SWE was performed in the same order at all measurements: VL, VM, and RF. VL was measured at about half way between the trochanter mayor and the lateral epicondyle of the knee (Coombes et al. 2018), VM at about one-third of the way between the medial epicondyle and anterior iliac crest (Coombes et al. 2018), and RF in the first-third distal between the proximal edge of the patella and anterior iliac crest (Ham et al. 2020). Care was taken to not put pressure on the skin, to avoid deformation of structures and muscle tissue (according to Kot et al. 2012).The ROI was maximized as much as possible, but excluding any aponeurosis. The transducer was aligned in plane with the fascicles and held in the same position during the whole process (according to Le Sant et al. 2017). The PRT test (as previously described) prior to the SWE was used as conditioning for the shear modulus testing, to guarantee the same muscle conditions. Participants were asked to remain completely relaxed during the measurements. Three videos of 15 s each were collected for each muscle. The mean of the five consecutive frames with the lowest standard deviation of the shear modulus averaged over the Range of interest (ROI) within a video was considered for further analysis. The two closest mean values per muscle from the three videos taken for each muscle were used to calculate the mean passive stiffness per muscle (Morales-Artacho et al. 2017).

#### Surface electromyography (EMG)

Muscle activity was monitored by EMG (myon 320, myon AG, Zurich, Switzerland) during the MVIC, PRT, and SWE measurements. After standard skin preparation, surface electrodes (Blue Sensor N, Ambu A/S, Ballerup, Denmark) were placed on the muscle belly of the VL, according to SENIAM recommendations (Hermens et al. 1999). The sample rate was 2000 Hz. EMG signals of the MVIC measurements were high-pass filtered (10 Hz, Butterworth) and the root-mean square (RMS, 50 ms window) values were calculated. The mean of 500 ms (± 250 ms around the peak value) was calculated around the maximum value. During the passive measurements we monitored the live EMG signal for activity. If signal changes were observed, the trial was repeated. Furthermore, a post-hoc analysis was performed for the PRT and SWE to ensure that the subject was relaxed, i.e. did not show EMG activity exceeding 5% of MVIC, if we could detect changes in the raw EMG signal during analyzing process (Gajdosik et al. 2005; Kato et al. 2010). In these cases, the EMG signal was high-pass filtered (10 Hz, Butterworth) and the root-mean square (RMS, 50 ms window) values were calculated.

#### Passive hip extension range of motion (ROM)

For the ROM measurements, a 3D-motion capture system (Qualisys, Göteborg, Sweden) was used. Eight cameras were used in fixed positions, and the system was calibrated with a standardized L-frame and wand at the beginning of each day. Reflective markers were added according the Qualisys Gait module (type: Cast) to the participant’s hip (with two extra markers on the lateral iliac crest to ensure a proper measurement in a supine position) and test leg. The participant was then asked to perform three modified Thomas tests of the test leg for 5 s each on a medical treatment bed. The participant lay supine, with the ischial tuberosity close to the edge of the bed (Younis Aslan et al. 2018). The participant was asked to hold the knees by hand, with the arm extended to ensure the same hip angle between measurements, and a flat lumbar spine. The legs were completely relaxed. While holding the contralateral leg in position, the test leg was lowered toward the floor and the participant was asked to remain as relaxed as possible in the end position. After processing the Qualisys data with Visual3D Professional (C-Motion, Inc., Germantown, USA), the angles of the joints were assessed. The attempt with the lowest hip extension angle was taken for further analysis.

#### Foam rolling intervention

**Fig. 2:**
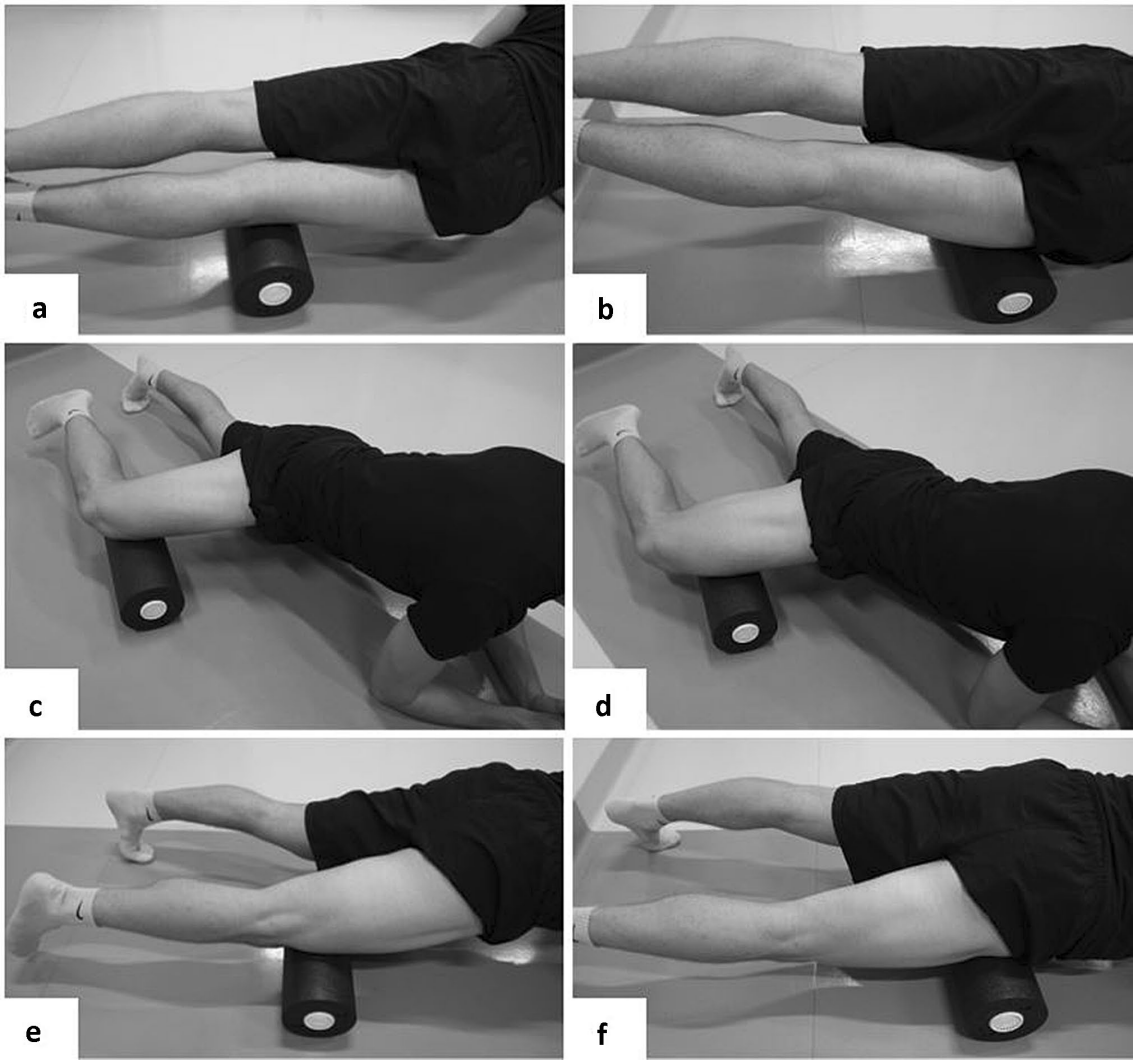
Start and turning positions during the foam rolling intervention: **a** start position for VL rolling; **b** proximal turning point during VL rolling; **c** start position for VM rolling; **d** proximal turning point for VM rolling; **e** start position for RF rolling; **f** proximal turning point for RF rolling

The same foam roller (Blackroll Booster Set in combination with a Blackroll Standard foam roll, Bottighofen, Switzerland) was used throughout the intervention. The vibration booster is an additional vibrating cylinder, which is positioned along the longitudinal hole in the middle of the foam roll. If switched on, the whole foam roll vibrates. The intensity of the vibration can be set to between 12 and 56 Hz in 15 different levels. The rolling was applied for 1 min per muscle, with a frequency of 30 repetitions per minute, including a break of 30 s between sets, resulting in an overall rolling duration of 180 s. The duration of 60 s per muscle of the thigh (VM, VL, RF) was chosen since Baumgart et al. (2019) reported a significant decrease of RF stiffness following 60 s of foam rolling on the RF muscle only. A metronome provided auditory signals to pace the movement, and the participant was asked to reach the starting position every 2 s (1 s from distal to proximal and 1 s from proximal to distal). Start position was always proximal to the knee (Fig. 2). The muscles in the right thigh only (test leg) were rolled in the following order: (1) VL (rolled on the lateral side of the thigh); (2) VM (rolled on the medial side of the thigh); and (3) RF (rolled on the anterior part of the thigh). During the VFR, the vibration booster was switched on with a vibration intensity of 32 Hz. During the NVFR, the same foam roll was used but the vibration mode was switched off. In both conditions, participants rolled with their own bodyweight and were asked to put as much pressure on the tissue as possible, to the point of discomfort.

### Statistical analyses

SPSS (version 26.0, SPSS Inc., Chicago, Illinois) was used for all the statistical analyses. To determine the intra-rater and inter-day reliability of the shear wave elastography measurements, intraclass correlation coefficients (ICC, 2-way mixed-effects model, absolute agreement definition) were used. The standard error of the mean of the shear modulus values was calculated as the standard deviation multiplied by the square root of one minus the ICC.

The variables tested were hip extension ROM, PRT, MVIC, and SWE of the VL, VM, and RF. A Shapiro–Wilk test was used to verify the normal distribution of all the variables. If the data were normally distributed, we performed a two-way repeated-measures ANOVA [factors: time (pre vs. post) and rolling modality (VFR vs. NVFR)]. Otherwise, we performed a Friedman test to test the effects of the foam rolling protocols (NVFR and VFR). If ANOVA with repeated measures or the Friedman test was significant, we performed a t test or a Wilcoxon test, respectively. To verify that the baseline conditions in VFR and NVFR were similar, paired t-tests or Wilcoxon tests were performed. To test possible differences between the rolling conditions (VFR vs. NVFR), paired t-tests or Wilcoxon tests of the delta values (post−pre) of each parameter were performed. The effect sizes d (for the t test) and r (for the Wilcoxon test) were established following the suggestions of Cohen (1988). Thus, the effect sized was defined as 0.2, 0.5, and 0.8 for a small, medium, and large effect, respectively. Moreover, the effect size r was defined as < 0.3, 0.3–0.5, and > 0.5 for a small, medium, and large effect, respectively. The alpha level was set to 0.05.

## Results

### SWE reliability and baseline measurement quality

The SWE ICC values between the pre-measurements of both test days (VFR vs. NVFR) for the VL, VM, and RF were 0.85, 0.94, and 0.84, respectively. Moreover, the standard errors of the mean values for the VL, VM, and RF were 0.609, 0.407, and 1.171, respectively. Baseline characteristics for the pre-measurements on both test days showed no significant difference in ROM (*P* = 0.09), PRT (*P* = 0.75), MVIC (*P* = 0.37), EMG (*P* = 0.15), VL shear modulus (*P* = 0.36), VM shear modulus (*P* = 0.74), and RF shear modulus (*P* = 0.06).

### Range of motion (ROM)

ANOVA for hip extension ROM revealed a significant time effect (*P* = 0.01; *F* = 7.45), no group effect (*P* = 0.89; *F* = 0.02), and a significant time × group interaction (*P* < 0.01; *F* = 11.71). Pairwise comparison of the hip extension ROM showed an increase in ROM in the vibration group (VFR; *P* < 0.01; *d* = 0.85), but not in the non-vibration group (NVFR; *P* = 0.51; *d* = 0.15) (see Table 1).

**Table 1:** Results of the maximum hip extension ROM, as well as the PRT, MVIC, shear modulus values for the VL, VM, and RF, and the MVIC-EMG-peak-values of the VL following NVFR (left) and VFR (right)

	NVFR		VFR	
	Pre	Post	Pre	Post
ROM hip (°)	− 1.0 ± 10.3	− 0.6 ± 9.1	1.0 ± 10.5	− 2.3* ± 9.6
PRT (Nm)	17.9 ± 2.9	17.5 ± 2.7	18.1 ± 2.4	17.6 ± 2.3
MVIC (Nm)	307.3 ± 51.8	315.9* ± 54.9	303.0 ± 58.3	317.2* ± 58.2
Shear modulus VL (kPa)	10.0 ± 1.6	10.2 ± 1.5	9.8 1.6	10.0 ± 1.4
Shear modulus VM (kPa)	9.0 ± 1.7	9.0 ± 1.6	9.0 ± 1.7	8.9 ± 1.5
Shear modulus RF (kPa)	9.5 ± 2.9	8.0* ± 1.8	10.4 ± 3.0	8.0* ± 2.3
MVIC-EMG-peak values (mV)	0.86 ± 0.59	0.86 ± 0.65	0.8 ± 0.5	0.83 ± 0.5

*MVIC* Maximum voluntary contraction, *PRT* passive resistive torque, shear modulus, *ROM* range of motion, *EMG* Electromyography

*Significant difference between pre- and post-session data, mean ± SD

### Passive resistive torque (PRT)

ANOVA for PRT revealed a significant time effect (*P* = 0.04; *F* = 4.60), but no interaction (*P* = 0.97; *F* = 0.00) or group effect (*P* = 0.67; *F* = 0.19). However, pairwise comparison of the PRT showed no changes in the vibration (VFR; *P* = 0.21; *d* = 0.30) or non-vibration group (NVFR; *P* = 0.11; *d* = 0.38) (see Table 1).

### Maximum voluntary isometric contraction (MVIC) peak torque

ANOVA for MVIC revealed a significant time effect (*P* < 0.01; *F* = 11.97), but no interaction (*P* = 0.28; *F* = 1.23) or group effect (*P* = 0.75; *F* = 0.11). The pairwise comparison showed an increase in both the vibration group (VFR; *P* = 0.01; *d* = 0.63) and the non-vibration group (NVFR; *P* = 0.02; *d* = 0.58) (see Table 1). Pairwise comparison of the delta values (post−pre) of the MVIC from the VFR and NVFR groups did not show a significant difference (*P* = 0.28; *d* = 0.24).

### EMG-analysis around the MVIC-peak-value

The Friedman test showed no significant effects on the MVIC-EMG values *(P* = 0.52; *χ*2 = 2.26). Moreover, the pairwise comparisons between pre and post values of the VFR and NVFR groups did not show significant differences (VFR – *P* = 0.65, *r* = − 0.13; NVFR – *P* = 0.60, *r* = − 0.15).

### Shear modulus values

The Friedman test showed a significant effect on the shear modulus of the RF (*P* < 0.01; *χ*2 = 19.29). The pairwise comparison showed a decrease in the shear modulus of the RF in both the vibration group (VFR; *P* < 0.01; *r* = − 0.78) and the non-vibration group (NVFR; *P* < 0.01; *r* = − 0.66). The pairwise comparison of the delta values (post − pre) of the shear modulus of the RF for the VFR and NVFR groups did not show a significant difference (*P* = 0.21; *r* = − 0.28). Moreover, ANOVA of the shear modulus of the VL and VM revealed no time effect (*P* = 0.24; *F* = 1.49, *P* = 0.62; *F* = 0.26), group effect (*P* = 0.18; *F* = 1.89, *P* = 0.60; *F* = 0.28), or interaction effect (*P* = 0.89; *F* = 0.02, *P* = 0.64; *F* = 0.22).

## Discussion

The purpose of this study was to compare the effects of VFR and NVFR applied for 3 min on the quadriceps muscle on muscle function (ROM, PRT, MVIC) and muscle mechanical properties (SWE of the VL, VM, and RF). Hip extension ROM increased in the VFR group only, while MVIC peak torque increased and shear modulus of the RF decreased in both groups (VFR, NVFR).

**Fig. 3:**
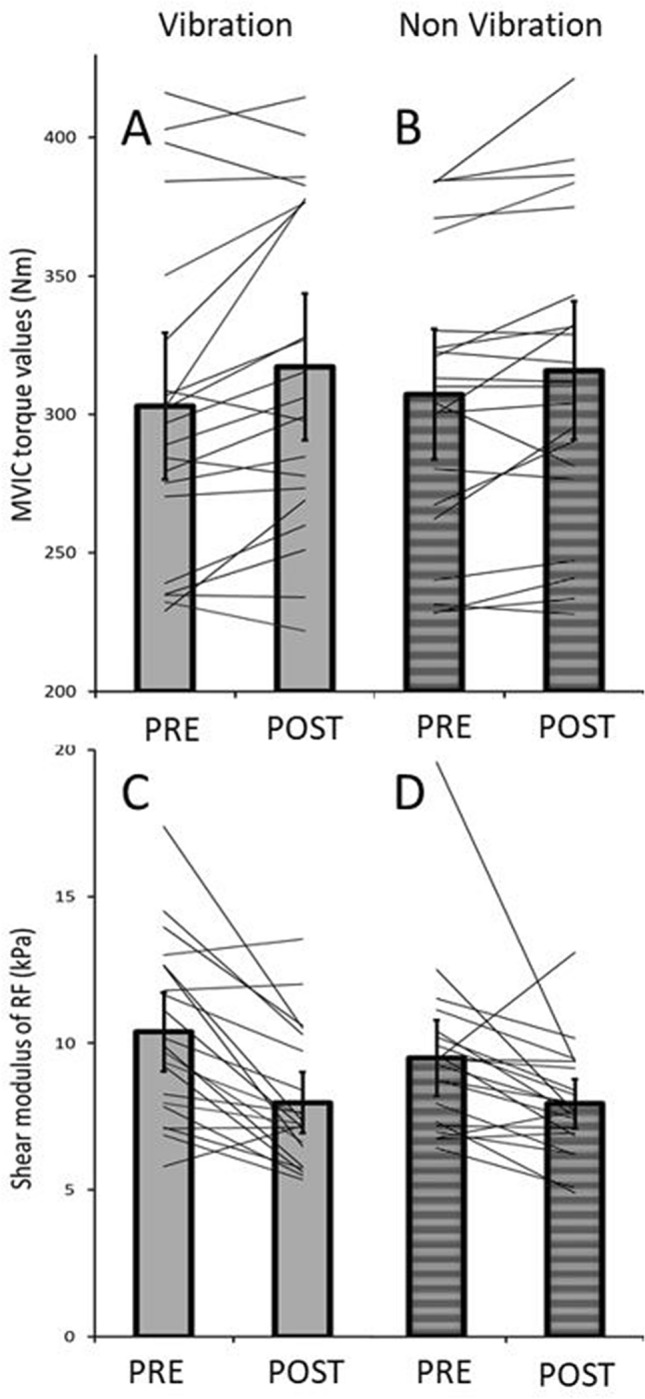
Individual changes in MVIC and shear modulus of the RF muscle after VFR and NVFR. **a** pre − post relations in MVIC with the VFR intervention; **b** pre − post relations with the NVFR intervention; **c** pre-post relations in shear modulus with the VFR intervention; **d** pre-post relations in shear modulus with the NVFR intervention

As in the current study, a superior effect for the increase in ROM following a single bout of foam rolling with VFR compared to NVFR was reported by Lim and Park (2019) and Lee et al. (2018) but not by other studies (Sağiroğlui 2017; Cheatham and Stull 2018; Lee et al. 2018; de Benito et al. 2019). A possible explanation for a greater ROM in VFR compared to NFVR might be found in a more advanced decrease in perception of pain (i.e. increased stretch tolerance). This has also been observed for whole-body vibration therapy (Veqar and Imtiyaz 2014) and localized vibration therapy (Germann et al. 2018). In addition, the superior effect of the pain modification of VFR compared to NVFR may be due to the greater contribution of the mechanoreceptors at higher vibration frequencies (Behm and Wilke 2019). This might be due to interstitial type III and IV receptors affecting the sympathetic and parasympathetic activity while responding to fast and sustained tension and pressure (Behm 2018; Behm and Wilke 2019). The result can be a more relaxed muscle due to a possible vasodilation, as a result of the regulation of heart rate, blood pressure, and ventilation, and also decreased pain sensitivity (Behm and Wilke 2019). An acutely increased ROM is mostly (Konrad et al. 2017) but not always (Magnusson et al. 1996) associated with a more compliant muscle–tendon unit by, for example, a decrease in PRT (and hence tissue stiffness) following a single static stretching exercise. Since foam rolling has been reported to have a similar magnitude of effect in increasing ROM as stretching (Wilke et al. 2020), the mechanical theory, i.e., the decrease in PRT is associated with an increase in ROM, might also be valid for an acute bout of foam rolling. Although we found a significant time effect for PRT, indicating an overall decrease in PRT due to foam rolling, the PRT changes in the individual groups did not reach significance [VFR (− 0.5 Nm; *P* = 0.21) and NVFR (− 0.47 Nm; *P* = 0.11)]. Since hip ROM increased following the VFR exercise, we would have also expected a meaningful decrease in PRT, indicating a more compliant quadriceps muscle–tendon unit. However, this was not the case in the present study. A probable explanation is that the PRT measurement in the toe region of the force–elongation curve did not induce enough stretch for the participants. This is in accordance with the findings of who reported a lack of changes in PRT in the toe region following a single stretching exercise. However, when the muscle–tendon unit is stretched until the linear region of the force–elongation curve, significant changes in PRT have been reported following stretching exercise (Kato et al. 2010). Thus, one could assume that a PRT measurement in a more stretched condition might have led to a significant reduction in PRT, at least following the VFR exercise. However, the shear modulus of the VM and VL did not change following both treatments (VFR and NVFR). Baumgart et al. (2019) showed a decrease in stiffness of the thigh muscle following 2 × 30 repetitions of NVFR applied to the anterior thigh. However, the authors used a myomechanographic device placed on the RF muscle. This approach allowed them to assess the stiffness of the thigh, which reflects the composite contributions of all the synergist muscles and precludes the measurement of the mechanical properties in individual muscles of the thigh. Regardless of the rolling technique (VFR or NVFR), we also found a decrease in the stiffness of the RF, but no changes in the stiffness of the VL and VM. A lack of change in VL stiffness (and also vastus intermedius muscle) was also reported following 5 × 45 s of NFVR of the lateral thigh by Mayer et al. (2019). Regarding the existing evidence about NFVR of the anterior thigh, there seems to be evidence that, within the leg extensors, the stiffness of the RF decreases while the VM, VL, and vastus intermedius are not affected. A possible explanation for why only the shear modulus of the RF in the current study changed in both groups (VFR and NVFR) might be found in the central location of the RF. In total, both groups (VFR and NVFR) completed three bouts of rolling for 60 s, starting the first bout with the focus on the VL, then the VM, and finally the RF. Considering the central placement of the RF, it is likely that the rolling of the VM and VL might have also affected the RF, leading to a greater intervention duration for this muscle. This might have induced the changes in muscle stiffness, while the VM and VL were unaffected in both groups (VFR and NVFR). With regard to the hamstring muscles, Morales-Artacho et al. (2017) reported a small decrease in overall hamstring stiffness following 5 × 1 min of NVFR. However, they did not report any changes in stiffness in the single muscles of the hamstrings. Moreover, it has been reported that NVFR of the calf muscle for 2 × 30 repetitions does not change its stiffness (Baumgart et al. 2019). To the best of our knowledge, the current study was the first to examine the effects on muscle stiffness following a bout of VFR. Furthermore, this study was the first to detect possible differences in muscle stiffness changes following VFR and NVFR. Although not significant, the tendency of a decrease in absolute values in RF stiffness following VFR was higher (− 7.23 kPa; − 23.2%) than after NVFR (− 4.68 kPa; − 16.4%) (see Fig. 3c + d). Furthermore, we found no changes in the shear modulus of the VL and VM following both VFR and NVFR. Surprisingly, changes in the shear modulus of the RF also occurred in the NFVR group, although there was no change in hip extension ROM. However, by a pairwise comparison of the overall stiffness of the quadriceps muscle by averaging the SWE values of the VL, VM, and RF, thigh stiffness decreased following VFR only (− 2.28 kPa; − 7.8%; *P* = 0.01), while following NVFR, the overall stiffness did not significantly change (− 1.34 kPa; − 4.7%; *P* = 0.10). This finding suggests that VFR [with a moderate to high magnitude of change (*d* = 0.59)] likely decreases thigh stiffness more than NVFR [with a small to moderate magnitude of change (*d* = 0.37)] (Cohen 1988). Furthermore, an advantageous effect of VFR in increasing ROM compared to NVFR might be the greater contribution of mechanoreceptors, due to the vibration (Behm and Wilke 2019).

In addition to the mechanical (i.e. decrease in PRT and hence decrease in muscle stiffness) and neurological (i.e. altered perception of stretch and pain) mechanisms which might explain the changes in ROM, other mechanisms such as thixotropic effects have to be considered with regard to the increase in ROM following VFR. Foam rolling induces pressure and friction on the treated muscle, skin, and fascia. This could have an impact on fluid viscosity and, hence, lead to less resistance to the movement of a joint (Behm 2018; Behm and Wilke 2019).

With regard to muscle performance, both treatments acutely increased knee extensor MVIC peak torque of the leg extensors. There is evidence that NVFR does not impair muscle force (Cheatham et al. 2015). Moreover, it has been suggested that vibration therapy can stimulate more muscle receptors (of all types), which leads to increased motor unit recruitment (Fallon and Macefield 2007; Germann et al. 2018). We hypothesized that VFR would have a superior effect in a possible increase in strength, compared to NVFR. Although not significantly different (*P* = 0.28), VFR showed a higher increase in MVIC (+ 14.2 Nm; + 4.7%) compared to the NVFR group (+ 8.6 Nm; + 2.8%) (see Fig. 3a + b). Sağiroğlui (2017) reported no differences in the effects between VFR and NVFR on countermovement jump performance. In addition, no differences between VFR and NFVR with regard to quadriceps strength was found by Lee et al. (2018). However, the same study reported a superior effect in knee flexion strength for VFR, but further study is necessary to obtain a clear picture. Monte and Zignoli (2021) found a positive relationship between active muscle stiffness assessed during short stretches and the rate of force development and belly gearing. Although active muscle stiffness and rate of force development differ from the parameters assessed in the present study, it could have been expected that changes in passive muscle stiffness/PRT after foam rolling would also affect force production. However, in the present study MVIC values increased after VFR and NVFR while PRT values did not change after any intervention. Furthermore, only shear modulus of the RF muscle, but not of VL or VM, decreased. Taking in account that the MVIC of the knee extensor muscles was assessed in a sitting position, the proportion of the RF, due to its unfavorable short length, might be too small to affect the peak force output. Therefore, the change of RF shear modulus might have a trivial effect on the force production in a knee extension. As the foam rolling interventions seem to have been not effective to decrease muscle stiffness or PRT, the findings from Monte and Zignoli (2021) cannot explain the increases in MVIC. An alternative explanation of the increased MVIC peak values in the knee extensor muscles after both interventions might be the warm-up effect of the whole-body during foam rolling. To stabilize the movement on the (vibrating) foam roll muscles in the whole body are activated and therefore blood flow increases and muscle temperature rises. This might lead to a better performance in general (Wiewelhove et al. 2019).

A possible limitation of the present study is the fast repetition duration of 1 s (time for a single roll in one direction along the chosen body part). This speed was chosen according to Halperin et al. (2014) who could find an increase in MVIC with the same rolling speed, which we could also detect in both groups (VFR and NVFR). Though, Behm et al. (2020) recommend a rolling duration of 2–4 s for rolling one direction of a chosen body part to reach the greatest ROM. The fast repetition duration might be a possible explanation for a missing change in hip extension ROM in the NVFR group. Another limitation of the study might be the short duration (~ 5 min) between warm-up and start of measuring mechanical properties which could have affected the ultrasound analysis because of intramuscular water and muscle temperature. However, this procedure was necessary to prepare the participants for the following MVIC and allowed standardized conditions for each participant and hence, has likely not influenced the outcome of this study. Please note, that MVIC data in the present study was not normalized for antagonist muscle contribution. However, we are confident that this has not influenced the results by expecting always the same amount of the antagonist muscle contribution during the MVIC throughout all measurements.

## Conclusion

We conclude that a 3-min rolling of the quadriceps muscles with VFR and NVFR can increase MVIC peak torque and has an impact on the shear modulus of the RF. However, we found that hip extension ROM increased only after VFR. Thus, for sports with flexibility (and possibly strength) demands, VFR might be the more efficient approach.

## Data Availability

All the data assessed within this study is presented in the manuscript

## Abbreviations

ANOVA: Analysis of variances
EMG: Electromyography
ICC: Intraclass correlation coefficient
MVIC: Maximal voluntary isometric contraction
NVFR: Non-vibration foam rolling
PRT: Passive resistive torque
RF: Rectus femoris
RMS: Root mean square
ROI: Range of Interest
ROM: Range of motion
SWE: Shear wave elastography
VFR: Vibration foam rolling
VL: Vastus lateralis
VM: Vastus medialis
